# Pharmit: interactive exploration of chemical space

**DOI:** 10.1093/nar/gkw287

**Published:** 2016-04-19

**Authors:** Jocelyn Sunseri, David Ryan Koes

**Affiliations:** Department of Computational and Systems Biology, University of Pittsburgh, 3501 Fifth Avenue, Pittsburgh, PA 15260, USA

## Abstract

Pharmit (http://pharmit.csb.pitt.edu) provides an online, interactive environment for the virtual screening of large compound databases using pharmacophores, molecular shape and energy minimization. Users can import, create and edit virtual screening queries in an interactive browser-based interface. Queries are specified in terms of a pharmacophore, a spatial arrangement of the essential features of an interaction, and molecular shape. Search results can be further ranked and filtered using energy minimization. In addition to a number of pre-built databases of popular compound libraries, users may submit their own compound libraries for screening. Pharmit uses state-of-the-art sub-linear algorithms to provide interactive screening of millions of compounds. Queries typically take a few seconds to a few minutes depending on their complexity. This allows users to iteratively refine their search during a single session. The easy access to large chemical datasets provided by Pharmit simplifies and accelerates structure-based drug design. Pharmit is available under a dual BSD/GPL open-source license.

## INTRODUCTION

There are a multitude of software packages and web services that assist in computer aided drug design (1), but a relative paucity of web services that support structure-based virtual screening. Those that exist, such as DockBlaster (2), iDrug (3), iStar (4), e-LEA3D (5) and MTiOpenScreen (6), are typically batch-processing services where the user submits a virtual screening job and receives the results hours or days later. They are also usually limited to screening a pre-determined library of compounds of limited size. Alternatively, advanced algorithms enable interactive time-scale searches, but existing web resources (7,8) are limited by a single search modality and a restricted search domain. In contrast, Pharmit provides both pharmacophore and molecular shape search modalities as well as ranking of results by energy minimization, and, in addition to providing a variety of pre-built compound libraries, allows users to upload their own compound libraries for screening.

Pharmit takes as its input a predefined pharmacophore query or can elucidate pharmacophore and shape queries from receptor and/or ligand structures. Structures may be provided by the user or extracted directly from the Protein Data Bank (PDB). Pharmacophore and/or molecular shape queries are created and edited in a modern interactive interface powered by 3Dmol.js (9), which provides high performance 3D molecular graphics without the need for plugins or Java. Once a query is defined, the user selects and searches a compound library for matching compounds. Results are typically returned in seconds and are displayed in-browser. A variety of filtering and ranking criteria can be applied, and hits can be further refined and ranked using energy minimization. Structure files of the query-optimized hit compounds can be downloaded, and the full session state can be saved and restored. In total, Pharmit provides a comprehensive online platform for structure-based virtual screening.

## COMPOUND LIBRARIES

**Figure 1.**
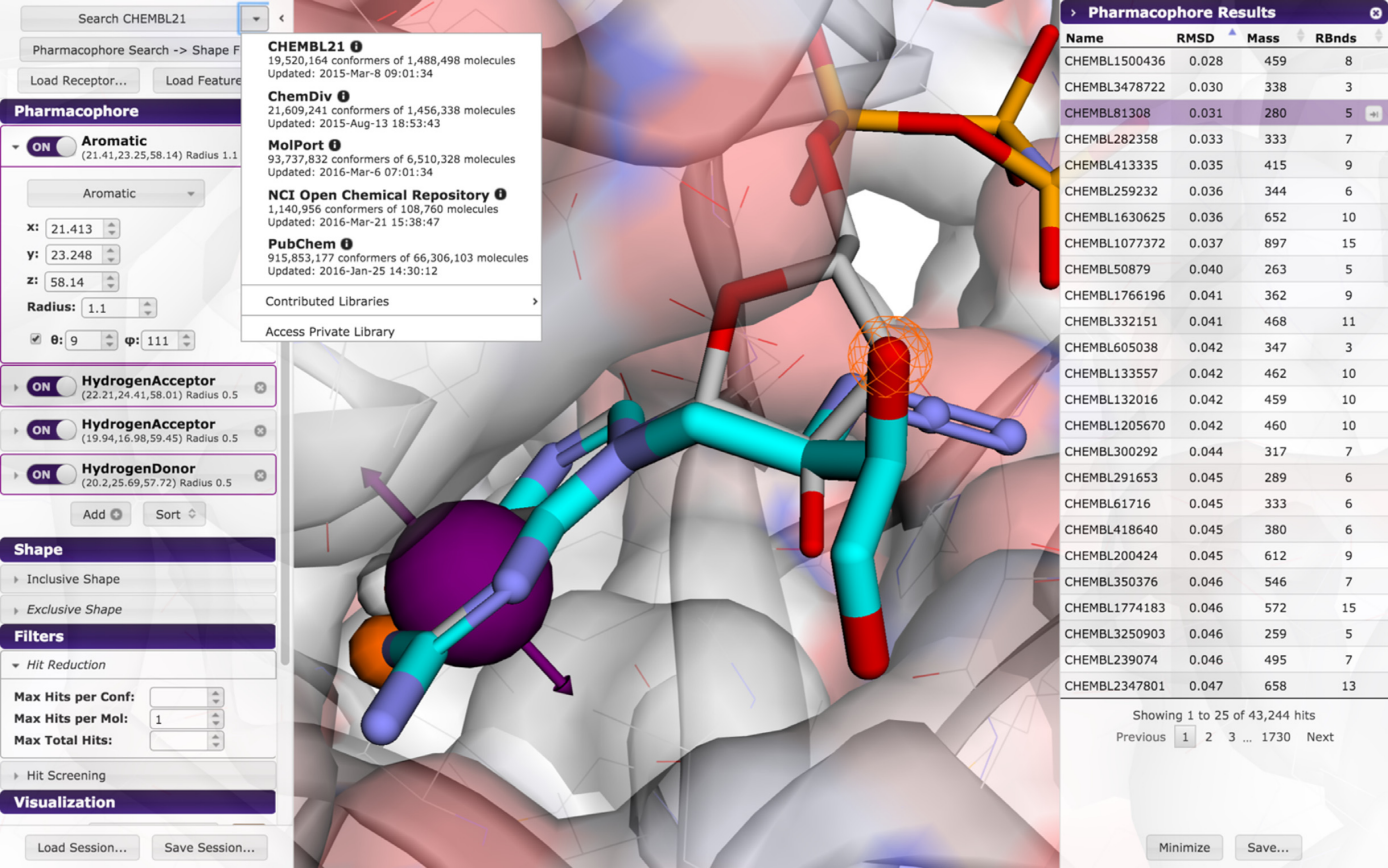
Pharmacophore as primary query. Each pharmacophore feature has a collapsible menu in the Pharmacophore panel (left) where its type, location, and radius, as well as number of atoms (for hydrophobic features) or directionality (if relevant) can be defined. Selected features are shown as solid spheres and unselected features as meshes. Filters may be set to reduce the number of hits by constraining the number of hits returned for a given conformer or molecule or the overall number of hits. Selecting a hit in the results panel (right) displays it, and its appearance can be adjusted in the visualization filter along with other aspects of the visual display. For example, here the query ligand is shown in light gray, and the selected hit compound is shown in cyan. This query against tyrosine-protein kinase c-Src (PDB 2SRC) is available on the Pharmit Examples page.

Unique to Pharmit is the ability to select from a number of provided compound libraries or to submit a custom library for screening. The library to screen is selected through a pull down menu in the search button (see Figure 1).

### Provided libraries

Large libraries corresponding to compound catalogs from a variety of sources are provided and periodically updated to ensure continued relevance, especially with regard to compound availability from commercial sources. Currently, Pharmit has pre-built libraries generated from CHEMBL21 (10), with >1.4 million compounds; ChemDiv (www.chemdiv.com), with >1.4 million compounds; MolPort (www.molport.com), with >6.5 million compounds; the NCI Open Chemical Repository (dtp.cancer.gov), with >108 000 compounds; and PubChem (11), with >66 million compounds. Although a search is limited to the compounds of the selected library, all compounds within these provided libraries are cross-annotated so, for example, it is possible to look up the PubChem record of a compound found by searching the commercial MolPort library to check for known bioactivities.

### Library creation

Users may submit their own libraries for screening. In the spirit of the open access and open-source nature of Pharmit, users are encouraged to make their submitted libraries publicly accessible, in which case they are available to all users for screening as a user contributed library. However, registered users have the ability to create a private library, as well as remove or update previously submitted libraries.

In order to create a library, compounds may be provided either in the two-dimensional SMILES or three-dimensional SDF formats. If the user uploads compounds in the SMILES format, duplicated canonical SMILES are removed, the molecules are protonated using OpenBabel (12) using default settings, and only the largest component of a molecule is retained (e.g. salts are removed). Then RDKit (rdkit.org) and the UFF force field (13) are used to generate up to ten 3D conformers for each compound resulting from this procedure. This approach has been shown to generate high quality conformations (14). Alternatively, if the user provides compounds in the SDF format, the provided structures are assumed to be valid conformers and are used directly, with protonation states determined by OpenBabel.

## QUERY DEFINITION

### Inputs

The typical starting point for a Pharmit session is a ligand-receptor complex structure, although a ligand-only structure or pharmacophore query file may be used as well. A Pharmit session can be automatically initialized using any complex in the PDB by inputing the corresponding PDB accession code on the Pharmit main page and selecting how active site water molecules should be treated (ignored, as part of the receptor, or as part of the ligand). The dropdown menu next to the box where a PDB code may be entered allows the user to select which ligand found in the PDB file should be used as the basis of the query.

Alternatively, a user can upload their own complex, in which case the receptor and ligand structures must be in separate files; these are uploaded by clicking ‘Enter Pharmit Search’ on the main page and then choosing ‘Load receptor’ and ‘Load features’ on the resulting page. Any file format supported by OpenBabel may be used. Note that the query ligand must be pre-positioned within the binding site of the receptor - Pharmit does not perform docking. Pharmit prepares the receptor by protonating it with OpenBabel, but no other modifications are made. Thus the user must decide whether there are missing residues that should be included, if the histidine protonation state is correct, or if any other structural changes to the receptor are necessary.

Pharmit can also initiate queries without a receptor, either in the form of a ligand structure or an externally generated pharmacophore query file, but in this case features that require a receptor, such as energy minimization, will not be available.

### Pharmacophore queries

A pharmacophore (15–17) defines the essential features of an interaction. Importantly, a pharmacophore includes the spatial arrangement of these features. Features supported by Pharmit include hydrogen bond acceptors and donors, negative and positive charges, aromatics, and hydrophobic features. As shown in Figure 1, a pharmacophore query specifies these features using tolerance spheres. Compounds match if they can be positioned so that their corresponding features are located within these spheres. Some features can have additional constraints, such as size (number of atoms) for hydrophobic features and direction for hydrogen bonds and aromatics.

Pharmit will identify all pharmacophore features present in a provided ligand structure. If a receptor structure is provided, it will identify which of these features are relevant to the protein-ligand interaction using distance cutoffs between corresponding features on the receptor and ligand (e.g. a hydrogen donor on the ligand and acceptor on the receptor). Only the interacting features will be enabled. Alternatively, a pharmacophore query can be initialized using pharmacophore files in MOE (18), LigBuilder (19), LigandScout (20), PharmaGist (21) or Pharmer (22) query formats. The features of the query can be interactively edited within the Pharmit interface as changes to the query editor are immediately reflected in the molecular viewer and clicking on a feature in the viewer selects it for editing, as shown in Figure 1.

### Shape queries

**Figure 2.**
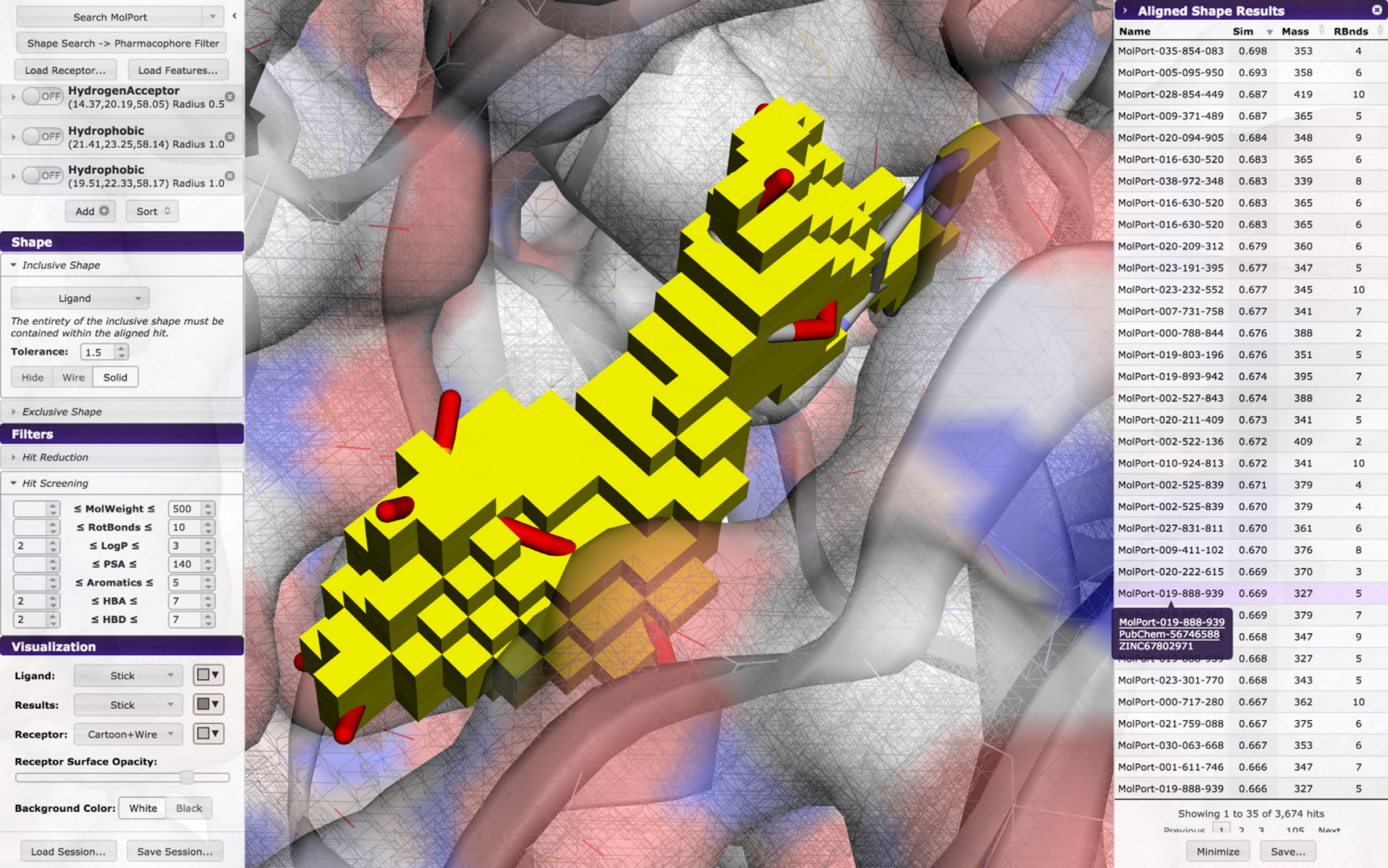
Shape as primary query. The shape menu defines inclusive and exclusive shape extent and tolerance as well as shape query visibility. Hits may be filtered by screening molecular properties relevant to their utility as drugs. Hovering over the name of a hit shows alternate molecular identifiers in all databases. This example uses tyrosine-protein kinase c-Src (PDB ID: 2SRC).

Similarity of molecular shape is a common method of structure-based virtual screening (23). Pharmit uses the Volumetric Aligned Molecular Shapes (VAMS) (24) method of shape search, which uses inclusive and exclusive shape constraints to identify matching molecular shapes. In Pharmit, inclusive constraints are specified using the shape of the provided ligand or by manually specified inclusion spheres. Inclusive constraints specify a minimum bound on the desired molecular shape; matching compounds will overlap these constraints. Exclusive constraints are specified using the shape of the provided receptor or by manually specified exclusion spheres. Exclusive constraints are used to limit the desired molecular shape; matching compounds are prohibited from overlapping these constraints. Both constraints are represented using voxelized volumes, as shown in Figure 2, and can be adjusted by growing or shrinking the constraint volume. A shape-first search with pharmacophore restraints is distinct from the commonly used ROCS with color shape method (25) as it does not optimize the position with respect to the pharmacophores.

## VISUALIZATION

The visualization scheme of the Pharmit interface is highly customizable. Both the color and representation style (e.g. sticks, spheres, cartoon, etc.) of the query receptor and ligand and the result compounds can be set individually as demonstrated in Figure 1. The transparency of the receptor surface, which is shown colored by electrostatic potential, may be adjusted from not present to fully opaque, and the background may be toggled between white and black. To facilitate inspection of molecular models, the query and results sidebars may be hidden to provide a full-window molecular viewer. Manipulation of the 3D viewer will be familiar to users of PyMol (26): left mouse button to rotate, right or scroll wheel to zoom, and middle button to translate.

## SEARCH

Pharmit provides two search modalities depending on whether the pharmacophore or shape query is the primary query. In both cases, the primary query determines the pose alignment of the hit compounds and the secondary query serves as an additional filter. If desired, the complete set of query-aligned results may be saved as a compressed SDF structure file.

### Pharmacophore/shape search

If the primary query is the pharmacophore query, the selected database is searched for compounds that match the specified pharmacophore using the Pharmer (22) search technology. Pharmer has comparable virtual screening performance to other pharmacophore methods (27) but is orders of magnitude faster.

Results are aligned to the pharmacophore to minimize the root mean squared deviation (RMSD) between the query features and the hit compound features. Results are sorted with respect to this pharmacophore RMSD. An example of a pharmacophore-oriented search is shown in Figure 1.

If a shape query is also present, then the shape constraints are applied to the pharmacophore-aligned pose; only heavy atom centers are compared to the shape constraints. That is, the pharmacophore-aligned results are filtered to ensure that hits have at least one heavy atom center that falls within the inclusive shape and no heavy atom center that falls within the exclusive shape. With this modality, the exclusive shape is generally the most useful, as it provides a way to eliminate compounds that match the pharmacophore but have significant steric clashes with the receptor. Due to the importance and increased specificity of the interactions specified by a pharmacophore, pharmacophore search followed by shape filtering is the default and recommended search modality.

### Shape/pharmacophore search

If the primary query is the shape query, VAMS (24) is used to search a shape index (28) for matches. Although VAMS does not exhibit as good virtual screening performance as more expensive methods, such as ROCS (25), it is orders of magnitude faster and serves as an effective shape-based pre-screen (24). With VAMS, molecules are pre-aligned to their moments of inertia and hits are aligned to the moments of inertia of the query. Molecular shapes are computed using the solvent excluded volume and are stored at a 0.5 Å resolution. A shape matches the query if, in its aligned position, the *entirety* of the inclusive shape constraint is contained within the shape while no part of the shape overlaps the exclusive shape. This is a more stringent requirement than that which is imposed when the shape query is used as a filter to a pharmacophore search. Matching compounds are sorted with respect to their Tanimoto shape similarity with the query ligand. An example of a shape-oriented search is shown in Figure 2.

If a pharmacophore query is present, it is used to filter the shape-aligned results. In this case, the pharmacophore features of the hit compound must fall within the tolerance spheres of the pharamacophore query with the compound in the shape-aligned pose. That is, a compound that would match a pharmacophore query if the query were the primary query may still be filtered out.

### Additional filters

Pharmit allows users to specify additional, non-structural filters on the results. As shown in Figure 1, the number of compounds returned can be reduced by restricting the number poses returned for every conformation, the number of conformations returned for every compound, and the total number of compounds.

Compounds may be filtered by specifying desired ranges for molecular properties that are commonly used to identify drug-like molecules. As shown in Figure 2, these include molecular weight, the number of rotatable bonds, log *P* (a measure of lipophilicity), topological polar surface area (indicative of the compound's ability to permeate cell membranes), the number of aromatic groups, the number of hydrogen bond acceptors and the number of hydrogen bond donors. Properties are precomputed using OpenBabel (12).

### Result browser

When a search is initiated, a results sidebar opens to display the search results, as seen in Figures 1 and 2. Results may be sorted in increasing or decreasing order based on RMSD (for pharmacophore searches), similarity score (for shape searches), molecular weight, or number of rotatable bonds. Clicking on a result displays the query-aligned compound in the molecular viewer, as Figure 1 shows. If desired, a new Pharmit session can be initiated from the ligand-protein complex represented by the selected hit compound. Compounds are annotated with all known database identifiers, as shown in Figure 2. Where applicable, these identifiers are linked to the source database, so it is possible to immediately investigate bioactivity data in PubChem or bring up the compound's order page from a commercial vendor.

## ENERGY MINIMIZATION

The query-aligned poses returned by pharmacophore and shape search are fully determined by the query. They are not influenced by interactions with the receptor. Furthermore, although the pose of the result is optimized to match the query, the conformation of the result is taken directly from the pre-generated conformers of the search database. Energy minimization of results optimizes both the pose and conformation of identified hits with respect to the provided receptor using the AutoDock Vina (29) scoring function and smina (30), a fork of AutoDock Vina with enhanced minimization functionality.

**Figure 3.**
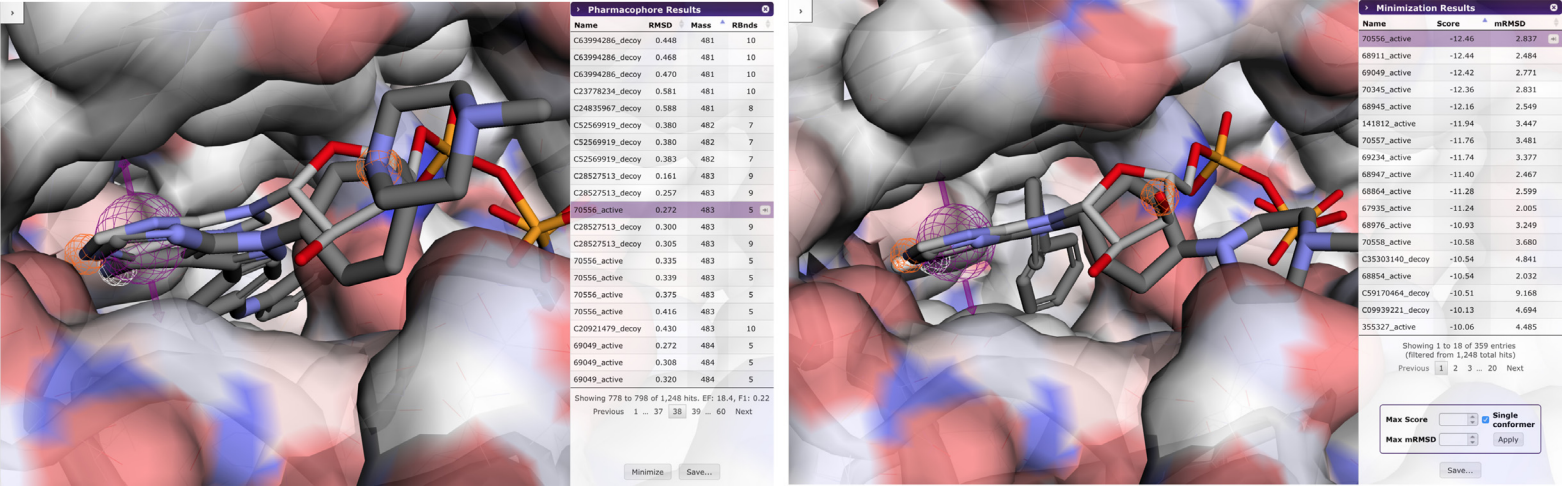
(Left) An example of a pharmacophore-aligned hit from the DUDe SRC target dataset for a query derived from PDB 2SRC. (Right) The same example after energy minimization. Steric clashes in the back pocket are resolved by rotating a phenyl resulting in an energy score of −12.46 kcal/mol.

Minimized compounds adopt a conformation at a local minimum identified by performing gradient descent on the energy surface starting from the initial query aligned pose. Results are sorted according to predicted binding affinity in kcal/mol (more negative values are more favorable). Also reported is a minimized RMSD (mRMSD) that is the RMSD between the query-aligned pose and the minimized pose. This provides an indication of how far the compound has deviated from the original query. Minimized results may be sorted and filtered by affinity and mRMSD to eliminate poses with unfavorable binding energies (e.g. >−6 kcal/mol) and significant deviations from the original query (e.g. 2 Å). An example of an energy minimized compound is shown in Figure 3 and contrasted with the unminimized pharmacophore aligned pose. The complete set of minimized compound structures can be saved, including scoring annotations, as a compressed SDF structure file.

## EXAMPLE

To demonstrate Pharmit's capabilities and typical usage, this section describes a virtual screen of Tyrosin-protein kinase C-SRC based off a complex (PDB 2SRC) with a nonhydrolyzable ATP analog (ANP). The resulting query is available as an interactive example from the Pharmit examples page. For this example we submitted the compounds of the the DUDe (31) SRC target benchmark as a contributed library. Compounds were renamed to include the keyword ‘active’ if they were active compounds, which allows Pharmit to automatically compute the enrichment factor (EF) and F1 score (geometric mean of recall and precision) of a search.

Beginning on the Pharmit main page, the user initiates a search by typing ‘2SRC’ into the ‘start from PDB’ box. This will retrieve the ligand names, ANP and PTR, from the PDB file, and they are displayed in the dropdown menu next to the PDB code box. The user then selects the ANP ligand and, for this example, chooses to ignore the binding site waters. After clicking ‘submit,’ the user will be taken to the main Pharmit interface. A set of interacting pharmacophore features will be automatically generated from the protein-ligand complex. The user then selects the DUDe SRC Benchmark from the Contributed Libraries list in the search selection menu. This contains 514 797 conformers of 35 024 molecules, of which 524 are known actives.

Pharmit identifies 26 pharmacophore features in ANP, 14 of which are interacting with the receptor. Searching with this default query yields no hits as it is overly specific. In general, we find that queries rarely benefit from having more than five or six distinct features. To generate a useful query for 2SRC, we rationally target the canonical hinge site, with which ANP strongly interacts, by including a hydrogen donor and acceptor and an aromatic ring to mimic the adenine moiety. Searching with this reduced query produces 92 398 hits (matching conformations) with an EF of 2.8. Adding a directionality constraint to the aromatic ring reduces the number of hits by two thousand and slightly increases the EF. The user can then interactively explore adding additional interacting features to the query as each query takes at most a few seconds. The addition of a hydrogen acceptor interaction on the ribose of the ligand generates 1,881 hits with an EF of 16.8 and produces the pharmacophore shown in Figure 1. The addition of an exclusive shape constraint with a tolerance of 2.5, which filters out compounds with severe steric clashes with the receptor, decreases the number of hits to 1248 and increases the EF to 18.4 as shown in Figure 3. Alternative queries can yield even higher enrichment factors at the expense of lower F1 scores due to fewer total active compounds being returned. In this case the user is guided by a benchmark library, but a similar interactive exploration is possible through rational investigation of the hit compounds generated by a query. Query results can be minimized to produce a more meaningful ranking, as shown in Figure 3, where the top 13 compounds, and 16 of the top 20, are correctly identified actives.

## DISCUSSION

Pharmit represents a significant advance over existing browser-based virtual screening servers. Among its virtues are its search speed, which is on the order of seconds; its high-resolution, smoothly animated visual interface; its customizability, including the capability to create custom compound libraries; and its expansive suite of search features, including an array of chemical constraints that allow the user to rapidly identify hits that are also promising drug candidates. It democratizes structure-based computer-aided drug discovery by offering free and open access to state-of-the-art software for exploring chemical space, making it ideal for research and education alike. Educators can deploy a high quality product to illustrate fundamental concepts in physical chemistry. Researchers can adapt the project to incorporate it into their existing drug discovery workflows. In settings where intellectual property protection is an issue, the entirely open source nature of Pharmit allows it to be deployed locally by the user, ensuring the complete privacy of queries and results. Pharmit anticipates the needs of the medicinal chemist by facilitating direct comparison between a search ligand and hits, permitting in-browser energy minimization, and enabling the user to rapidly launch new sessions using molecules found via a query. Pharmit enables the user to perform structure-based virtual screening in a manner that is fast and intuitive.
